# Mitochondrial transplantation: opportunities and challenges in the treatment of obesity, diabetes, and nonalcoholic fatty liver disease

**DOI:** 10.1186/s12967-022-03693-0

**Published:** 2022-10-22

**Authors:** Yifei Chen, Fuji Yang, Ying Chu, Zhihua Yun, Yongmin Yan, Jianhua Jin

**Affiliations:** 1 Department of Laboratory Medicine, Wujin Hospital Affiliated With Jiangsu University (The Wujin Clinical College of Xuzhou Medical University), Changzhou, 213017 Jiangsu Province China; 2 School of Medicine, Jiangsu University, ZhenjiangJiangsu Province, 212013 China; 3 Central Laboratory, Wujin Hospital Affiliated With Jiangsu University (The Wujin Clinical College of Xuzhou Medical University), Changzhou, 213017 Jiangsu Province China; 4 Department of Oncology, Wujin Hospital Affiliated With Jiangsu University (The Wujin Clinical College of Xuzhou Medical University), Changzhou, 213017 Jiangsu Province China

**Keywords:** Diabetes, Extracellular vesicles, Mitochondrial transfer, Nonalcoholic fatty liver disease, Obesity

## Abstract

Metabolic diseases, including obesity, diabetes, and nonalcoholic fatty liver disease (NAFLD), are rising in both incidence and prevalence and remain a major global health and socioeconomic burden in the twenty-first century. Despite an increasing understanding of these diseases, the lack of effective treatments remains an ongoing challenge. Mitochondria are key players in intracellular energy production, calcium homeostasis, signaling, and apoptosis. Emerging evidence shows that mitochondrial dysfunction participates in the pathogeneses of metabolic diseases. Exogenous supplementation with healthy mitochondria is emerging as a promising therapeutic approach to treating these diseases. This article reviews recent advances in the use of mitochondrial transplantation therapy (MRT) in such treatment.

## Introduction

Metabolic diseases, including obesity, diabetes, and nonalcoholic fatty liver disease (NAFLD), occur worldwide, with increasing incidence and prevalence. In 2015, an estimated 604 million adults had obesity and 414 million people had diabetes [1]. The incidence of diabetes is expected to rise to 629 million by 2045. Approximately 85% of all patients with type 2 diabetes (T2D) are either overweight or obese [2]. NAFLD is tightly linked to obesity. Furthermore, 25% of the world’s population might have NAFLD [3]. Mitochondrial-dysfunction–related oxidative stress (OS), insulin resistance (IR), and metabolic disorders are important contributing factors in the development of obesity, diabetes, and NAFLD [4, 5]. Therefore, the repair of mitochondrial homeostasis is expected to produce a potential therapeutic effect on metabolic diseases and their complications. Existing studies indicate that mitochondrial transfer restores the bioenergetics of damaged mammalian cells via actin-based tunneling nanotubes (TNTs), extracellular vesicles (EVs), cell fusion, and extrusion [6–8]. In 2017, a human clinical study first reported using isolated mitochondria to treat cardiomyopathy as an innovative strategy for improving mitochondrial dysfunction [9]. In this review, we summarize the advances of mitochondrial transplantation therapy (MRT) in metabolic-disease treatment to provide valuable insight into this scope.

Notably, the clinical application of MRT is still limited. More research is needed to accelerate the development of and access to mitochondrial drugs to optimize them for the benefit of patients. We found that MRT is challenged by the immune response. In addition, we discuss methods of maintaining mitochondrial stability in mitochondrial drug products. Finally, we summarize delivery strategies for healthy mitochondria and predict the future clinical application of MRT.

## Function of mitochondrial transfer in intercellular communication

Mitochondrial bioenergy is necessary for cell survival [10]. Mitochondrial dysfunction has been observed in a variety of diseases, including T2D, NAFLD, aging, cancer, cardiovascular diseases, and degenerative brain diseases [11]. Regulation of mitochondrial biology and function could potentially be used to treat various diseases caused by mitochondrial damage. Treatment strategies for mitochondrial dysfunction are generally divided into the following categories: (i) increasing mitochondrial biogenesis; (ii) reducing dysfunctional mitochondria and replacing them with active ones; (iii) delivering or replacing dysfunctional components; (iv) intervening in the consequences of mitochondrial dysfunction; and (v) reprogramming the mitochondrial genome [12, 13]. However, to date, almost none of these strategies have yielded satisfactory results. The main problems include methods to identify suitable targets and a lack of a reliable method to target damaged mitochondria. Recently, the field of MRT has received increasing attention as an innovative strategy for treating mitochondrial diseases by replacing disabled mitochondria.

Mitochondria are considered to be retained in cells for their lifetimes. The transfer of mitochondria between cells has not yet been confirmed. In 1982, Clark and Shay first demonstrated that isolated mitochondria with mutant genes for chloramphenicol could naturally transfer antibiotic resistance to susceptible cells [14]. In 2006, Spees et al*.* reported evidence of mitochondrial transfer between mammalian cells [15]. So far, mitochondrial transfer has been found between different types of cells, such as mesenchymal stem cells (MSCs) and alveolar cells, astrocytes and neurons, and different bone marrow mesenchymal stem cells (BM-MSCs) [16, 17].

## Mode of mitochondrial transfer

**Fig. 1:**
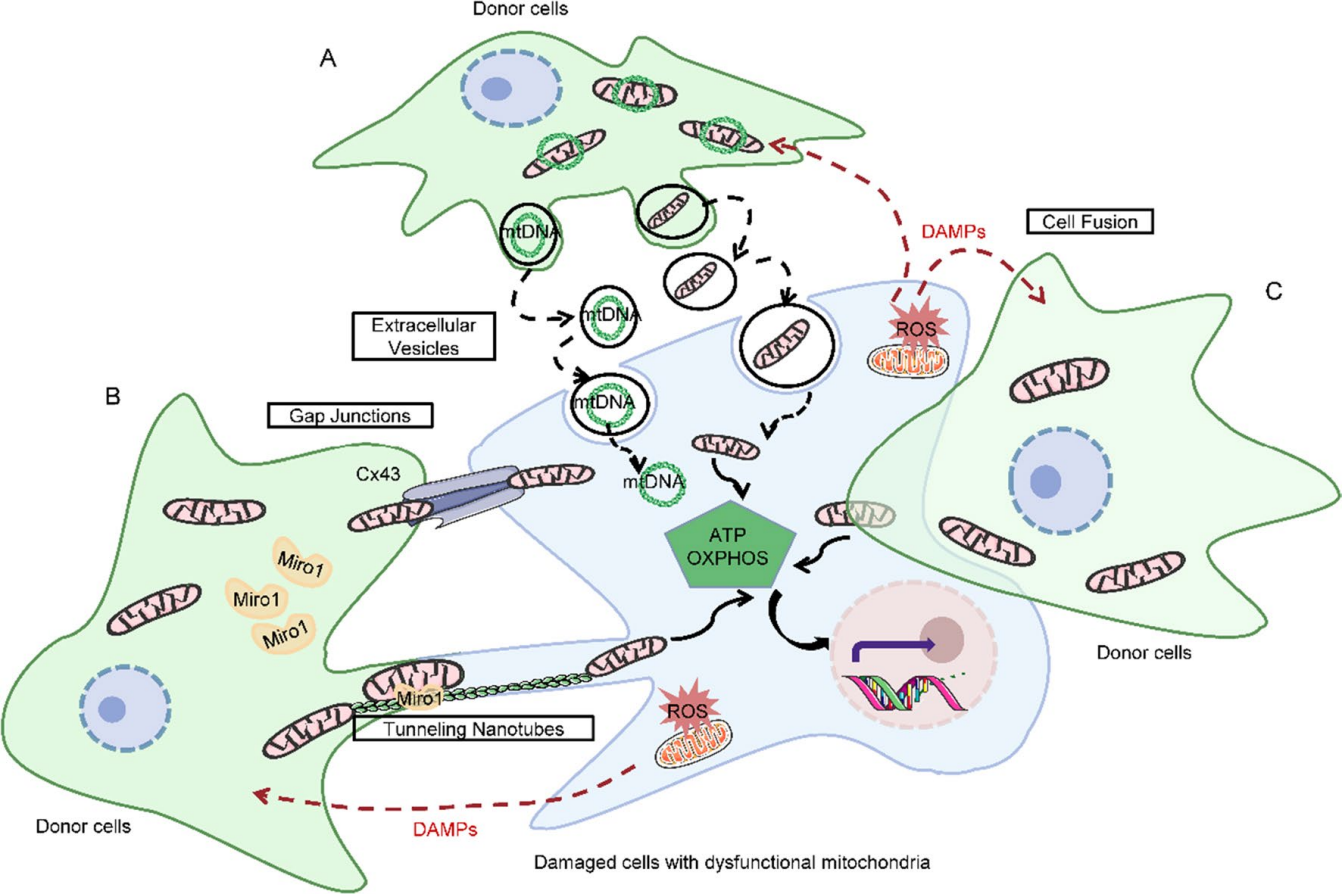
Schematic representation of the various mitochondrial-transfer modes utilized between donor cells and damaged cells with dysfunctional mitochondria. **A** Extracellular vesicles (EVs) can convey mtDNA or fragments of mitochondria; though less well documented, microvesicles are suggested to contain entire mitochondria. **B** Cx43-mediated gap junctions serve at the cell–cell junction to enable mitochondrial transfer. Tunneling nanotubes (TNTs) are actin-dependent cytoskeletal protrusions that also serve as cytoplasmic bridges between cells. Miro1 regulates the transport of mitochondria across TNTs. C. Cell fusion enables sharing of cytoplasmic contents during either transient or permanent fusion of the plasma membranes of two cells

Mitochondrial transfer relies on communication between a donor cell and a recipient cell and can be regulated by several structures, such as TNTs or EVs [18–21] (Fig. 1). TNTs conduct transcellular mitochondrial transfer from adjacent healthy cells to rescue recipient cells from a bioenergetic deficit [22], and this process might be bidirectional [23]. EVs have also been shown to transfer integral mitochondria [24]. The sharing of EV-derived mitochondria has been observed in different cell types [25–27]. In addition, cell-to-cell transfer of mitochondria can also be conducted via gap junctions (GJs), cell fusion, and direct uptake of isolated naked mitochondria [28, 29]. The occurrence of mitochondrial transfer is considered a new category of intercellular signaling and is involved in multiple pathophysiological conditions [30]. This phenomenon of cell-to-cell mitochondrial transfer could be a new approach to the treatment of mitochondrial diseases by replacing non-functional mitochondria in damaged tissues or cells with functional ones.

### Mitochondrial transfer via TNTs

Several structures mediate cell-to-cell mitochondrial transfer [31]; TNTs are the major ones. Rustom et al*.* originally discovered TNTs as a new intercellular communication channel [19]. TNTs are thin, tubular, F-actin–based structures 50–700 nm in diameter that can connect cells over long distances. They can transport cellular organelles such as mitochondria, lipid droplets, proteins, and micro–ribonucleic acids (miRNAs) [32]; such transfer can be unidirectional [33] or bidirectional [23].

Some of the proteins involved in the formation of TNTs are small GTPase (Miro1), connexin 43 (C×43), M-Sec (also known as tumor necrosis factor alpha [TNF-α]–inducible protein 2), the exocyst complex, and leukocyte-specific transcript 1 (LST1). Miro1 is essential for mitochondrial transport. MSCs with Miro1 overexpression promoted mitochondrial–alveolar epithelial-cell (EC) transfer in a co-culture system [34]. In mouse models of lung injury and asthma, intravenous (i.v.) injection of MSCs that overexpressed Miro1 partially reversed ischemic effects and improved neurological function compared with injection of unmodified MSCs [16]. C×43 is an important regulator of TNT formation through intercellular GJ channels (GJCs) for mitochondrial transfer [35]. In addition, the exocyst complex regulated by M-Sec is also required for TNT formation [36, 37]. LST1 is a transmembrane protein that can recruit RalA to the submembrane region and accelerate the interaction between RalA and the exocyst complex. Meanwhile, LST1 recruits the actin cross-linked protein filamin. The combination of LST1, M-Sec, RalA, and the exocyst complex promotes remodeling and cross-linking of actin filaments, leading to cell membrane protrusion and fusion and ultimately to TNT formation [38]. However, the mechanism underlying the fusion of the protruded section of the membrane with target cells remains unclear.

### Mitochondrial transfer via extracellular vesicles

EVs are membrane-shed vesicles 50–1000 nm in diameter [7]. They can carry a variety of bioactive cargos, including organelles, membrane proteins, and small molecules. Therefore, EVs play important roles in mitochondrial or mitochondrial-DNA (mtDNA) transcellular transfer in certain types of cells [39]. In 2012, Islam et al*.* [17] first showed that BMSC-derived EVs could carry mitochondria to damaged alveolar ECs in a lipopolysaccharide (LPS)–induced acute lung injury (ALI) model. Similarly, MSCs facilitate the phagocytic activity of macrophages through mitochondria-containing EVs in clinically relevant models of lung injury [40]. This hypothesis has gained significant support from recent studies on ECs, immune cells, astrocytes, and neurons [41, 42].

Compared with TNTs, the mechanism by which EVs mediate mitochondrial transfer remains unclear. The nicotinamide adenine dinucleotide–positive (NAD^+^)/Cluster of Differentiation 38 (CD38)/cADPR/Ca^2+^ pathway might contribute to EV-mediated mitochondrial transcellular transfer. Intracellular NAD^+^ increases and transfers to the extracellular environment under stress conditions in glioma cells; this leads to an increase in intracellular Ca^2+^ concentration via the NAD^+^/CD38/cyclic adenosine diphosphate ribose (cADPR)/Ca2 pathway following remodeling of the actin cytoskeleton and invagination of the cell membrane, thereby completing endocytosis of EVs. Nevertheless, inhibition of endocytosis reduces the mitochondrial transfer of BMSCs to damaged lung ECs [43]. Similarly, mitochondrial transcellular transfer by EVs between astrocytes and neurons has also been shown to depend on the NAD^+/^CD38/cADPR/Ca^2+^ pathway [27, 44].

### Mitochondrial extrusion and cell fusion

TNTs, EVs, and C×43 GJCs represent the main routes that mediate mitochondrial transcellular transfer. However, some other routes exist, such as cytoplasmic fusion and mitochondrial extrusion [45]. Cytoplasmic fusion is a common phenomenon in which the membranes of two or more cells fuse to share organelles [8]. Rearrangement of the actin cytoskeleton and fusion of glycoproteins on both cell membranes are required for cell fusion [46]. Cell fusion results in massive mitochondrial delivery into recipient cells. Elongation of the distance between cells leads to the transfer of fewer mitochondria [47]. Evidence shows that cell fusion can modulate the potency of stem cells; mitochondrial transfer is crucial for stem cells in regeneration and tumorigenesis [31].

Naked mitochondria or mitochondrial components can also be extruded by exocytosis and internalized by endocytosis without a carrier. In a mouse model, Boudreau et al. demonstrated that platelet-derived naked mitochondria are released into the extracellular environment [48]. In addition, cells sensitive to chloramphenicol (CAP) and efrapeptin (EF) were found to endocytose naked mitochondria isolated from CAP- and EF-resistant fibroblasts [14] to recover the viability and bioenergy of recipient cells [49].

## Metabolic diseases and mitochondrial dysfunction

Metabolic diseases involve multiple cell types, tissues, organs, inflammatory signaling cascades, and humoral factors [50, 51]. Disruption of mitochondrial function is a common feature of inherited metabolic diseases, including diabetes, obesity, and NAFLD.

### Mitochondrial dysfunction in diabetes and obesity

T2D is the fourth-leading cause of death worldwide. Its prevalence has greatly increased due to the adoption of sedentary lifestyles [52]; it is predicted that approximately 642 million adults worldwide will suffer from diabetes by 2040 [53].

**Fig. 2:**
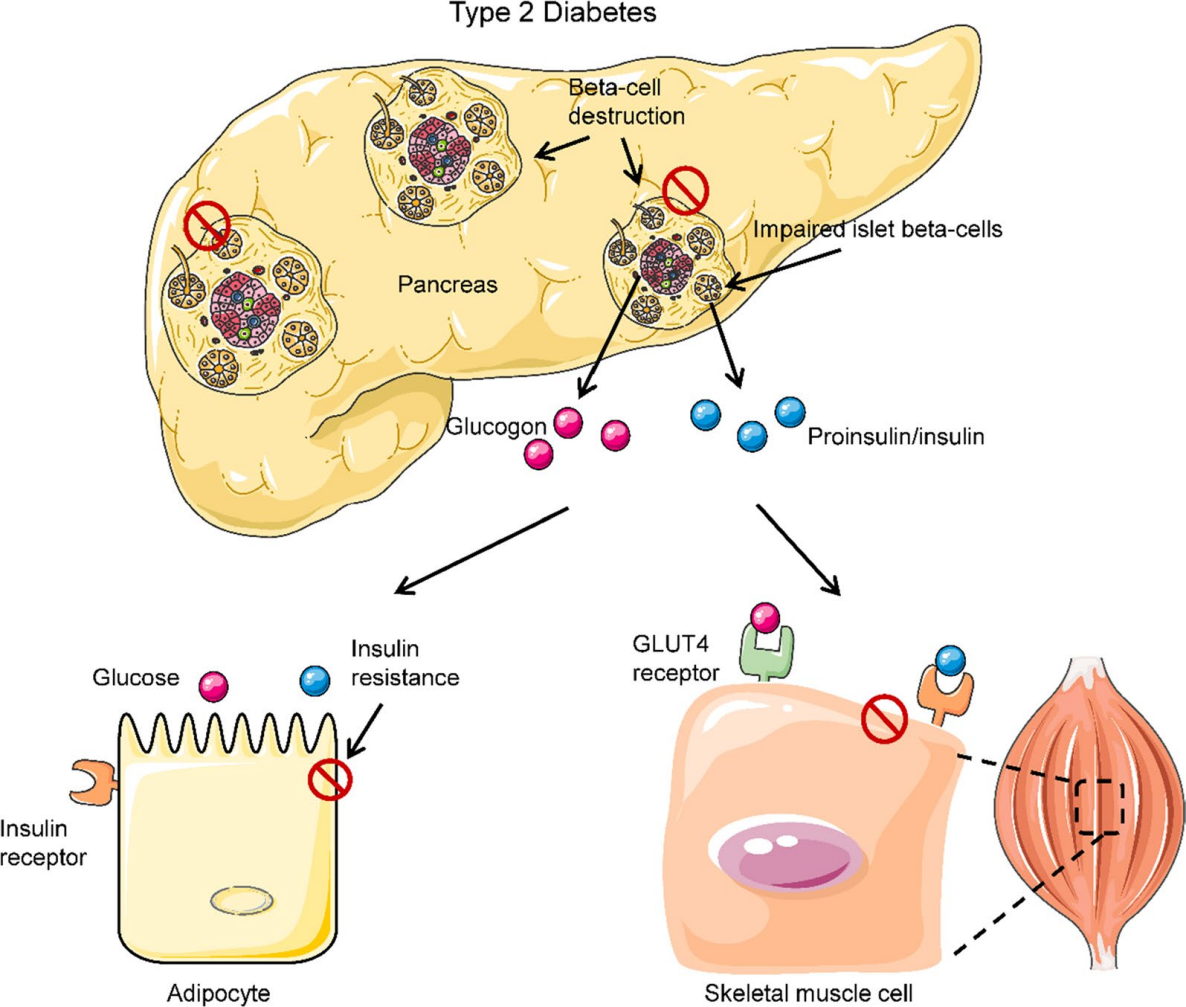
Comparison of healthy and type 2 diabetic phenotypes. In individuals with T2D, islet β-cells undergo apoptosis, and the function of the surviving cells is impaired, which results in markedly reduced insulin levels in circulation. Additionally, peripheral-tissue insulin resistance impairs the action of insulin, resulting in reduced uptake of glucose from circulation as a result of decreased GLUT4 translocation to the membrane. Reduced insulin levels result in hyperglycemia and hyperlipidemia, and subsequent T2D-associated symptoms manifest. Inappropriate glucagon secretion, diminished incretin hormone action, increased proinsulin secretion, impaired pancreatic-islet neural regulation, and islet amyloid deposition are also characteristics of T2D

T2D exhibits pancreatic β-cell dysfunction and enhanced pancreatic α-cell function, which causes chronic hyperglycemia induced by peripheral-tissue IR [54] (Fig. 2). First, pancreatic β-cell dysfunction results in absolute or relative insufficiency of insulin secretion and poor peripheral glucose and fat uptake, which leads to hyperglycemia and dyslipidemia. Second, hyperglycemia and hyperlipidemia activate pancreatic α-cells via increased glucagon production. Recent studies point to peripheral-tissue IR, as a sensitive reaction in prediabetic individuals, as a prerequisite to the development of T2D [55, 56]. IR occurs due to an imbalance between the supply and demand of nutrients in multiple tissues, including skeletal muscle, liver, and adipose tissue [57]. It is also associated with mitochondrial dysfunction [58]. In addition, mitochondrial dysfunction contributes greatly to age-dependent IR [59] and induces diabetic microvascular (cardiomyopathy, nephropathy, retinopathy, and neuropathy) [60–63] and macrovascular (myocardial ischemia) [64] complications. Therefore, mitochondrial quality control is a promising intervention for managing T2D and obesity.

As a leading cause of disability and death, T2D represents a major cost to healthcare systems. Emerging evidence suggests that mitochondrial dysfunction precedes the development of T2D [65]. Although the role of mitochondria in regulating OS, IR, and insulin secretion has been widely recognized, the effect of mitochondrial dysfunction on T2D is still a mystery.

Mitochondria are a major source of reactive oxygen species (ROS), which are critical to redox homeostasis, metabolism, and multiple cellular functions through apoptosis and maintenance of Ca^2+^ levels in patients with diabetic cardiomyopathy [66–68]. Many studies suggest that IR is closely associated with ROS production [65]. Nicotinamide adenine dinucleotide phosphate (NADPH) and mitochondrial-oxidase–derived ROS (mROS) can block serine kinase signaling for insulin receptor substrate 1 (IRS-1) protein phosphorylation [69] and impair insulin signaling [70]. In addition, mROS can ameliorate the disorder of the electron transport chain (ETC) dominated by complex I [71] and significantly reduce adenosine triphosphate (ATP) synthesis in pancreatic-islet β-cells [72]. In contrast, another report demonstrates that mROS causes IR without affecting insulin signaling or components of the mitochondrial respiratory chain [73]. Interestingly, mROS can activate mitochondrial fission [74]. Mitochondrial fission is often associated with mitochondrial-membrane potential (Δψ_mt_) depolarization [75], which damages the ability of mitochondrial Ca^2+^ signaling to control mitochondrial motility [76]. Additionally, the dynamic interplay between Ca^2+^ release, mitochondrial motility, and mitochondrial Ca^2+^ uptake forms the basis for a homeostatic mechanism for mitochondrial distribution and calcium signaling. However, the mechanism by which mitochondrial fission generates IR has not yet been elucidated and is worthy of attention.

Mitochondrial dysfunction and IR might have a bidirectional effect, which would affect the control of blood glucose levels in the body through different mechanisms such as glycogenolysis, glucose uptake, and gluconeogenesis [77]. In patients with IR, aerobic exercise enhances insulin action by stimulating mitochondrial biogenesis and function [78]. In addition to increased glucose uptake, insulin stimulates mitochondrial activity in adipocytes [79] and skeletal muscle [80], which can significantly reduce hepatic lipid accumulation and enhance insulin sensitivity and glucose homeostasis [81]. Conversely, poor mitochondrial function reduces insulin sensitivity [82]. Spontaneous mutations of mitochondrial deoxyribonucleic acid (DNA) caused by aging or environmental factors inhibit mitochondrial β-oxidation [83], which increases fatty acid accumulation and induces IR by inhibiting glucose transporter 4 (GLUT4) translocation. Certain DNA mutations lead to alteration in oxidative phosphorylation (OXPHOS), resulting in less ATP production for glucose transport and thereby increasing IR [84]. However, whether a direct relationship exists between mitochondrial dysfunction and IR remains unclear. More importantly, whether mitochondrial capacity is a cause or consequence of IR remains unknown. Despite numerous studies on the subject, it is still uncertain whether mitochondrial dysfunction is connected to a common signal that promotes IR. Further studies and specific animal models are necessary for a better understanding of the process by which mitochondrial dysfunction produces IR.

Mitochondria might be the master regulators of insulin secretion [85, 86]. Reduced nicotinamide adenine dinucleotide (NADH) cytoplasmic mitochondrial shuttling [87], mitochondrial Ca2 + signaling [88], mROS [89], and tricarboxylic acid (TCA) metabolic intermediates [90] can promote insulin secretion. A question that has intrigued us and other researchers in the field in which mitochondrial bioenergetic state is more beneficial for increased glucose sensitivity, including the ability of peripheral tissues to take up and use glucose, and subsequent insulin release in diabetic patients. Since both excessive mitochondrial fission and fusion can lead to changes in insulin secretion, maintenance of the ideal balance between mitochondrial fusion and fission appears to be a promising therapeutic target.

### Mitochondrial dysfunction in NAFLD

NAFLD, defined as excessive accumulation of fat in the liver [91], has a global prevalence of 30% [92]. With the prevalence of obesity and metabolic syndrome, NAFLD is considered an important cause of chronic liver disease in developed countries and regions such as Europe, the United States, and affluent areas of China. In addition, NAFLD is involved in the occurrence of T2D, which seriously affects patients’ quality of life (QoL). However, the underlying mechanism of NAFLD remains unknown and many therapies have no good effect on it [93].

**Fig. 3:**
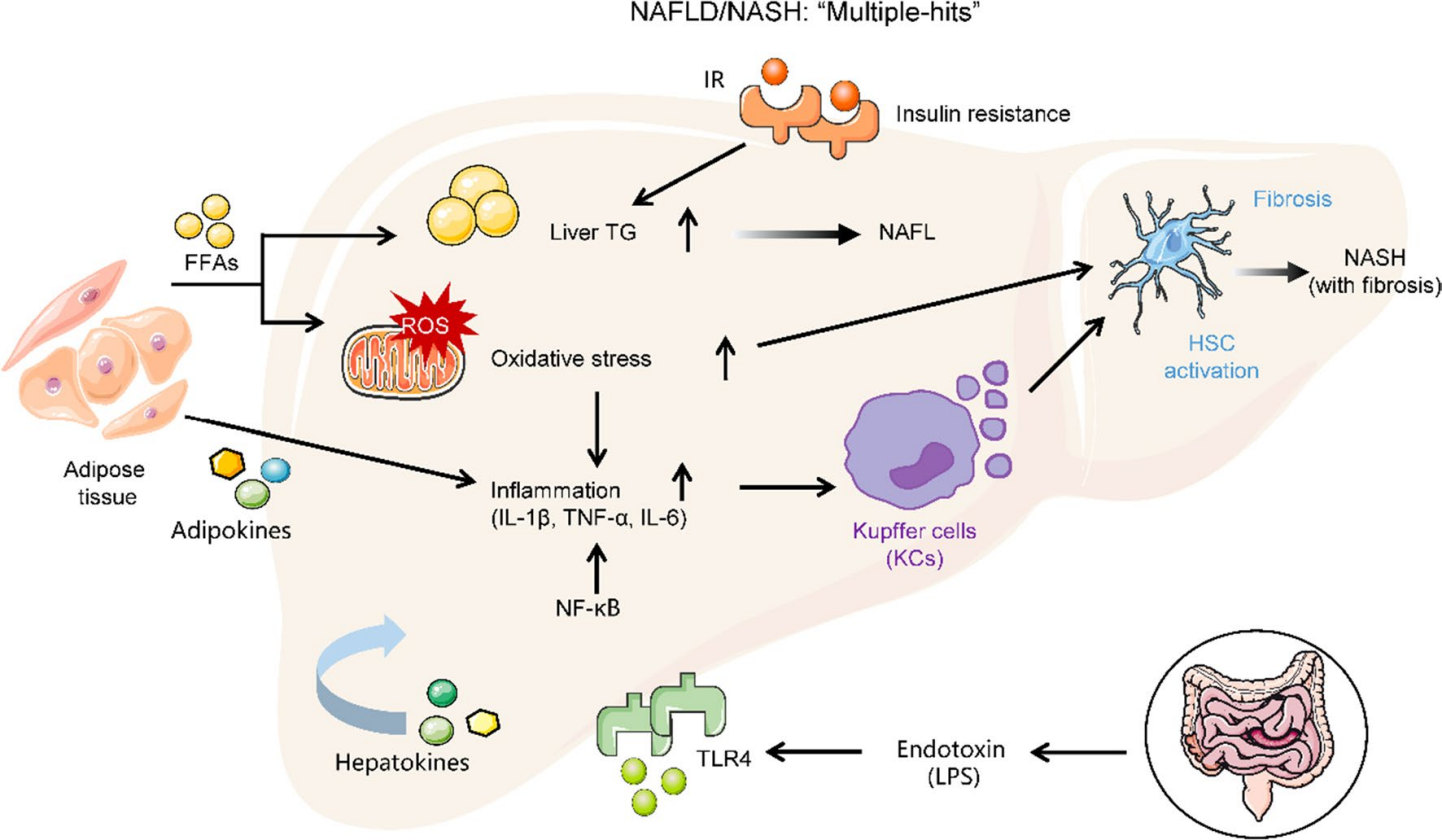
Multiple-hit pathogenesis of nonalcoholic fatty liver disease (NAFLD). NAFLD begins with hepatic lipid accumulation and insulin resistance and progresses to nonalcoholic steatohepatitis (NASH) with the collaboration of various factors such as inflammation, endotoxin, organokines (adipokines and hepatokines), and oxidative stress

NAFLD is driven by multiple parallel factors, such as OS, hepatic inflammation, free fatty acid (FFA), IR, and dysfunction of adipose tissue [94] (Fig. 3). In a high-fat diet (HFD) mouse model, abnormal ETC exhibited high levels of mROS, which increased OS [95] and promoted the development of NAFLD [96]. Excessive OS further aggravates stellate-cell activation via lipid peroxidation (oxidation of FFA) and liver inflammation (cytokine release) [97]. Furthermore, lipid peroxidation indirectly accelerates inflammation, which inhibits insulin receptor signaling and the insulin sensitivity of the liver, thereby inducing steatosis and fibrosis [98, 99]. Lipid peroxidation also leads to lipotoxicity and dysfunction of adipose tissue [99]. In a NAFLD mouse model, while mitochondrial dysfunction occurred concurrently with incomplete fatty acid β-oxidation [100], the decline in such β-oxidation resulted in accumulation of inflammation and IR, suggesting a link between mitochondrial dysfunction and fatty liver diseases. This finding indicates that mitochondria-targeting medicines could have important implications for NAFLD patients.

NAFLD is considered a mitochondrial disease [101]. In the liver, mitochondria participate in many vital physiological processes, particularly in energy metabolism [102–104]. Sirtuins (SIRT) comprise a family of nicotinamide adenine dinucleotide (NAD +)-dependent lysine deacylases that regulate the life span, aging, and metabolism [105]. The mitochondrial sirtuins are involved in metabolic regulation and antioxidative defense. In contrast to other members of SIRT, the enzymatic activities of SIRT4 have remained unclear. Emerging evidence show that circulating levels of sirtuin 4, a potential marker of oxidative metabolism, related to coronary artery disease in obese patients suffering from NAFLD, with normal or slightly increased liver enzymes [106]. In patients with NAFLD, hepatic mitochondria exhibit ultrastructural lesions and decrease the activity of the respiratory-chain complexes; this decrease results in the accumulation of ROS to form lipid peroxidation products, which in turn causes hepatitis and necrosis [107]. Studies have shown that mROS can also induce activation of hepatic stellate cells into myofibroblasts to generate collagen via transforming growth factor β (TGF-β) produced by Kupffer cells, thus further promoting the development of NAFLD into liver fibrosis and cirrhosis [108]. In addition, disorders of mitochondrial function can lead to the accumulation of FFA in hepatocytes, subsequently causing IR in an energy crisis [109, 110]. In the liver, IR typically results in the upregulation of mitochondrial function in hepatocytes [111]. In contrast, in adipose and cardiac tissues, IR impairs mitochondrial respiratory function in adipocytes and cardiomyocytes [112], suggesting a complex tissue specificity between insulin sensitivity and energy metabolism. Interestingly, hypertrophy-induced IR sensitizes hepatocytes to mitochondrial dysfunction and oxidative damage, which subsequently exacerbates inflammation and hepatic stem cell (HSC) activation and aggravates liver damage [113]. Therefore, IR induced by different factors in NAFLD warrants further research.

The role of OS and hepatic IR in inducing NAFLD can be explained by the role of mitochondrial dysfunction in hepatic lipid metabolism [114]. Therefore, pharmacological therapies that target mitochondria could be promising for NAFLD intervention. Ailing Fu et al*.* injected naked mitochondria from human hepatocytes with hepatocellular carcinoma (Hep G2 cells) into the livers of mice with fatty livers and found that this treatment could improve energy production in the liver and reduce hepatic lipid accumulation and oxidative damage to restore hepatocytic activity; this provides new insights into preventing the progression of NAFLD [115]. We firmly believe that MRT is potentially an optimal therapeutic approach to a variety of mitochondrial diseases.

## Mitochondrial transfer in metabolic diseases

The development of a reliable mitochondrial-delivery protocol is a major challenge that must be addressed before widespread clinical application of MRT is possible. Due to the immunogenicity of isolated mitochondria [116–118], the cell-mediated mitochondrial transfer is considered a more promising therapeutic strategy (Table 1).

**Table 1:** Researches of mitochondrial transplantation in obesity, diabetes and hepatic diseases

Mitochondrial source	Recipient	Therapeutic outcome	Mechanism	References
Bone marrow derived mesenchymal stem cell	Renal proximal tubular epithelial cells (streptozotocin- induced diabetic)	Suppressing ROS production and inhibited apoptosis of PTECs	Gap junctions	[119]
Bone marrow derived mesenchymal stem cell	HFD-induced T2DM-associated NAFLD	Combat NAFLD via rescuing dysfunction mitochondria	Cell fusion	[120]
Mesenchymal stem cells (MSCs)	Macrophages	Alleviates kidney injury in diabetic nephropathy mice	Tunneling nanotube (TNT)	[121]
Adipose MSCs	Human islet β-cells	Improves islet insulin secretory function	TNT	[122]
Platelets	Hippocampal neurons (db/db mice with Diabetes-associated cognitive impairment)	Attenuates oxidative stress and neuronal apoptosis	Mitochondrial transplantation	[123]
Adipocytes	White adipose tissue (WAT) macrophages of HFD-induced obese mice	Reduces energy expenditure and exacerbates diet-induced obesity	Undefined	[124]
Adipocytes	Cardiomyocytes with acute oxidative stress injured	Limits cardiac ischemia/reperfusion injury	Small extracellular vesicles (sEVs)	[125]
Macrophages	Brown adipose tissue	Ensure efficient thermogenesis in brown adipose tissue	Extracellular vesicles (EVs)	[126]
Cardiomyocytes	Cardiomyocytes (pregestational diabetes mellitus (PGDM)-exposed, HFD-diet-exposed)	Reduce cardiomyocytes apoptosis and boost respiratory	Mitochondrial transplantation	[127]
Tissue samples (Zucker lean rats)	Hearts from zucker diabetic fatty (ZDF fa/fa) rats	Enhances myocardial postischaemic function and decreases myocellular injury	Mitochondrial transplantation	[128]
Human hepatoma cells (HepG2 cells)	High-fat diet (HFD)-induced mouse fatty liver	Rescue of hepatocyte mitochondrial function	Endocytosis (naked mitochondria)	[115]

*NAFLD* Nonalcoholic fatty liver disease; *T2DM* type 2 diabetes mellitus; *ROS* reactive oxygen species

MSCs have been widely investigated in regenerative medicine and drug delivery due to their low immunogenicity and targeting properties. In streptozotocin (STZ)–induced diabetic animals, mitochondrial dysfunction in hyperglycemia-induced proximal-tubule ECs (PTECs) is alleviated by BMSCs via transfer of mitochondria. The direct administration of isolated mitochondria under the renal capsule of STZ rats generates a rapid improvement of PTEC morphology and in the structures of the tubular basement membrane and brush border. In vitro, mitochondria isolated from BMSCs enhance the expression of mitochondrial superoxide dismutase 2 (*SOD2*) and B-cell lymphoma 2 (*Bcl-2*) and reduce the production of ROS. Furthermore, the administration of isolated mitochondria can decrease PTEC apoptosis by reducing the nuclear translocation of receptor-activated receptor γ-coactivator-1α (PGC-1α) and upregulating the expression of megalin and sodium-glucose cotransporter-2 (SGLT2) [119]. These findings provide new insights into the mechanism by which BMSCs exert therapeutic effects on diabetic nephropathy (DN). Similarly, in an HFD-induced NAFLD model, mitochondrial transfer from BMSCs to steatotic cells was observed to reduce fat accumulation. The recipient steatotic cells markedly enhanced liver function, OXPHOS activity, ATP production, and Δψ_mt_ while reducing ROS levels, weight gain, steatosis, and disturbances in glucose and lipid metabolism in obese mice [120]. In contrast, umbilical cord MSCs have been shown to have clinical promise due to their accessibility, expandability, and multipotentiality. In DN, umbilical-cord MSCs alleviate renal injury by promoting the polarization of macrophages to an anti-inflammatory phenotype, which depends on PGC-1α–mediated mitochondrial biogenesis and PGC-1α/transcription factor EB (TFEB)–mediated lysosomal autophagy [121]. In addition, studies indicate that human adipose–derived MSCs mediate mitochondrial transfer to pancreatic islets, which improves the insulin secretion function of the islets [122]. Nevertheless, MSCs are challenged by uncertainties in culture method, source cells, expansion level, storage and transportation conditions, and ethical approval, which hinder their clinical application. Platelets are considered a more attractive source for autologous mitochondrial transplantation due to their abundance, accessibility, and low immunogenicity. In a DB/DB diabetes-associated cognitive impairment (DACI) mouse model, lateral ventricular injection of platelet-derived mitochondria (Mito-Plt) attenuated cognitive impairment and mitochondrial dysfunction. Mechanistically, Mito-Plt injected into the lateral ventricle were internalized into hippocampal neurons, which contributed to the recovery of mitochondrial function, mitigation of OS, and neuronal apoptosis and reduced Aβ and Tau accumulation in the hippocampus [123].

Recent studies have highlighted that cell-to-cell mitochondrial transfer contributes to metabolic diseases. In adipocyte-specific mitochondrial-reporter mice, macrophages have been found to acquire endogenous mitochondria from neighboring adipocytes. Further genome-wide clustered regularly interspaced short palindromic repeats (CRISPR)–CRISPR-associated protein 9 (Cas9) knockdown revealed that heparan sulfate (HS) is critical for mitochondrial uptake in macrophages [124]. White adipose tissue (WAT) macrophages exhibit lower HS levels, resulting in reduced intercellular mitochondrial transfer from adipocytes to macrophages in mice with obesity or deletion of the myeloid-cell HS biosynthesis gene exostosin-1 (*Ext1*), which increases body weight and obesity and decreases glucose tolerance and insulin sensitivity [125]. Recent findings have demonstrated that thermochemically stressed brown adipocytes can release EVs containing oxidatively damaged mitochondrial parts to avoid failure of the thermogenic program. When reuptaken by parental brown adipocytes, mitochondrial-derived EVs reduce PGC signaling and levels of mitochondrial proteins, including uncoupling protein 1 (UCP1). Depletion of macrophages in vivo causes abnormal accumulation of extracellular mitochondrial vesicles in brown adipose tissue (BAT), impairing the thermogenic response to cold exposure [126]. This current research contributes to a better understanding of how macrophages interact with adipocytes and could guide treating obesity. In addition, adipocytes can release EVs to protect cardiomyocytes from acute OS. EVs containing adipocyte-damaging mitochondrial granules enter the blood circulation and are taken up by cardiomyocytes, thereby triggering an increase in ROS and inhibiting ischemia/reperfusion (I/R) injury to mouse hearts via compensatory antioxidant signaling in the heart [127].

Several studies have used isolated mitochondria from various sources as an intervention in many diseases. In a diabetic cardiac I/R rat model, injection of naked mitochondria enhanced myocardial function by improving ATP content and myocardial viability after ischemic perfusion [128, 129]. Meanwhile, in NAFLD mice, serum transaminase activity was decreased after intravenous administration of exogenous mitochondria, which consequently reduced lipid accumulation and oxidative damage in mice with fatty livers. MRT offers unique therapeutic potential for the amelioration of NAFLD [115] and, in conclusion, has a bright future in metabolic-disease therapy.

## Problems in mitochondrial transfer

### Ethical issues

The debate over whether mitochondrial replacement therapy (MRT) should be allowed is based on scientific and ethical issues. How to classify MRT as a medical procedure is uncertain. Mitochondrial donation involves the transfer of genetic material but not of nuclear genetic material, which leads to the question of whether it should be conceptualized as ova or tissue donation [130]. Numerous studies have shown that personal characteristics are determined entirely by nuclear DNA121, suggesting that a child’s physical characteristics come from its parents, not from the mitochondrial donor. Therefore, MRT is more likely to be classified as similar to tissue donation. Nonetheless, while MRT has great potential to meet standards of safety and therapeutic efficiency, it requires thorough ethical analysis before clinical implementation can be considered. One central issue is the origin of donor mitochondria, including autografts, allografts, and xenografts. Each donor source can have its unique ethical and biological implications. In autologous transplantation, mitochondria from tissues with a lower risk of mtDNA mutation can be used to treat a highly compromised organ of the same person. This donor source rarely raises ethical concerns, but it entails great biological challenges that require further complex experimentation and the use of animal models to verify. All transfers would use mitochondrial donations from genetically close family members. Ideally, the human donor and recipient should share the same haplotype [131]. Alternatively, if no close relatives are available, haplotype matching could be considered [132]. Another ethically tricky option for all transfers is the potential use of still-viable mitochondria from a dead human relative in treatment [133, 134].

While MRT raises many ethical and safety concerns, it may also offer new therapeutic options. The United Kingdom (UK) has recently taken an important step toward allowing and regulating the use of MRT therapy by developing relevant guidelines [135]. This was passed in October 2015 under the license and regulations of the UK Human Fertilization and Embryology Authority (HFEA) [136]. This medical and legal advance opens up a range of possibilities for families with severe mitochondrial diseases to have their own genetically healthy children.

### Immunological reaction of transplanted mitochondria

A few reports to date have discussed immune responses during MRT. Understanding the responsible mechanism would be valuable in reducing the risks associated with MRT. For acquired mitochondrial disease, transplantation of mitochondria-derived from autologous cells without inflammation and autoimmune responses appears feasible. McCully et al*.* have shown that autologous mitochondrial transplantation induces no immune response in various animal models [137]. In a rabbit model of ischemic cardiomyopathy, a single injection of autologous mitochondria isolated from pectoralis major tissue failed to increase various inflammatory markers in serum or the production of anti-mitochondrial antibodies [138]. Likewise, in a porcine I/R model, serum cytokine levels did not increase significantly after a single autologous mitochondrial transplantation [139].

In the case of congenital mitochondrial disease, autologous mitochondrial transplantation might not be suitable, because mitochondria in other tissues might be dysfunctional. To tackle this issue, a discussion of autoimmune responses generated by allogeneic mitochondrial transplantation is critical. Ramirez Barbieri et al*.* [140]. investigated immune response and damage-associated molecular patterns (DAMPs) in mice following single or multiple intraperitoneal injections of allogeneic mitochondria and found that serum cytokine and mtDNA levels did not increase after either autologous or allogeneic mitochondrial injection. In contrast, Brennan et al. [141] showed that the heart develops a marked rejection response early in a single injection of allogeneic mitochondria. Activation of vascular ECs by extracellular mitochondria accelerates graft rejection. Elevated secretion of inflammatory cytokines and chemokines by activated vascular ECs increases T-cell adhesion and penetration into allograft tissues. Another study reports that the blood of organ transplant donors is enriched in mitochondria-derived DAMPs, which leads to the elevation of pro-inflammatory cytokines and chemokines during donor transplantation [142]. To expand the potential and stability of MRT, further research into and resolution of challenges such as post-transplant immune responses are required.

## Mitochondrial source, purity, and storage

The mitochondrial-donor screening system, mature mitochondrial-function evaluation, and mitochondrial-preservation methods determine the stability of mitochondria as a pharmaceutical product for clinical applications. Therefore, sufficient improvements in MRT strategy for better treatment of patients depend on sufficient mitochondrial reserves.

The source of mitochondria is critical for MRT and is influenced by metabolic profile, age, health of the source organ, ease of access to organelles, and histocompatibility. Mitochondrial bioenergetics express differently in different organs. The numbers of mitochondria in muscle, brain, and adipose tissue are the same, but mitochondria in muscle have higher Δψ_mt_ [143, 144]. Due to its high regenerative potential, easy access, organelle density, and relative OXPHOS coupling index, the liver is considered a good source of donor mitochondria [145]. A recent study has shown that donor tissue age correlates with mitochondrial mass [146]; mitochondria from young healthy mice with higher Δψ_mt_ and antioxidant capacity are better able to suppress tumor cell proliferation than mitochondria from older mice. However, the relevance of histocompatibility to MRT remains uncertain.

Another issue is the quality of the isolated mitochondria. Intact functional mitochondria are essential for successful MRT, although possibly only certain mitochondrial components might play beneficial roles [147]. A high-quality mitochondrion should at least have outer- and inner-membrane integrity, the ability to phosphorylate ADP in vitro, and high expression of oxidative coupling factors [145] so that it can be used in MRT. Furthermore, cytochrome C (cyt c) in respiration assays or activity assays of specific enzymes in the mitochondrial matrix is recommended to control organelle integrity. Recommended oxygen consumption in high-quality mitochondrial respiration induced by cyt c is no higher than 15%. In addition, no activity of matrix enzymes should be detectable in the supernatant of the organelles.

A storable preparation of mitochondria for clinical applications would be significantly beneficial for MRT, but it would require the establishment of a method that permits mitochondria to be stored for an extended period. The composition of the mitochondrial-storage solution is critical to maintaining mitochondrial activity in low-temperature storage. Both Eurocollins [148] and the University of Wisconsin (UW) [149] solutions have been developed for organ preservation. A study evaluating mitochondria isolated from rat livers found that UW solution can maintain cyt c content and complex II activity after storage for 24 h. However, when rat livers are stored in Eurocollins solution, glucose permeates the hepatocytes, which causes mitochondrial expansion and loss of complex III and IV activities due to the absence of antioxidants [150]. Geiger et al*.* report that mitochondrial-respiration capacity is maintained above 80% after cold storage for 24 h in 4-(2-hydroxyethyl)-1-piperazineethanesulfonic acid (HEPES)–sucrose-based buffer. A storage period exceeding 2 days leads to a substantial decrease in breathing ability. Compared with traditional mitochondrial-reservation solutions, Nukala et al. [151] explored the possibility of storing isolated mitochondria in dimethyl sulfoxide (DMSO); they found that mitochondrial OXPHOS capacity remained unchanged when mitochondria were stored frozen in 10% DMSO, but mitochondrial activity was reduced. These results indicated that storing mitochondria in a refrigerator in any preservation buffer has limited ability to protect them from damage. Therefore, preservation solutions used in mitochondrial cold storage must be optimized to maintain mitochondrial structure and function. The addition of antioxidants is effective in maintaining complex III and IV activity and the addition of colloids is effective in maintaining the mitochondrial structure, which could be useful for such optimization.

## Strategies for mitochondrial-transfer optimization

**Fig. 4:**
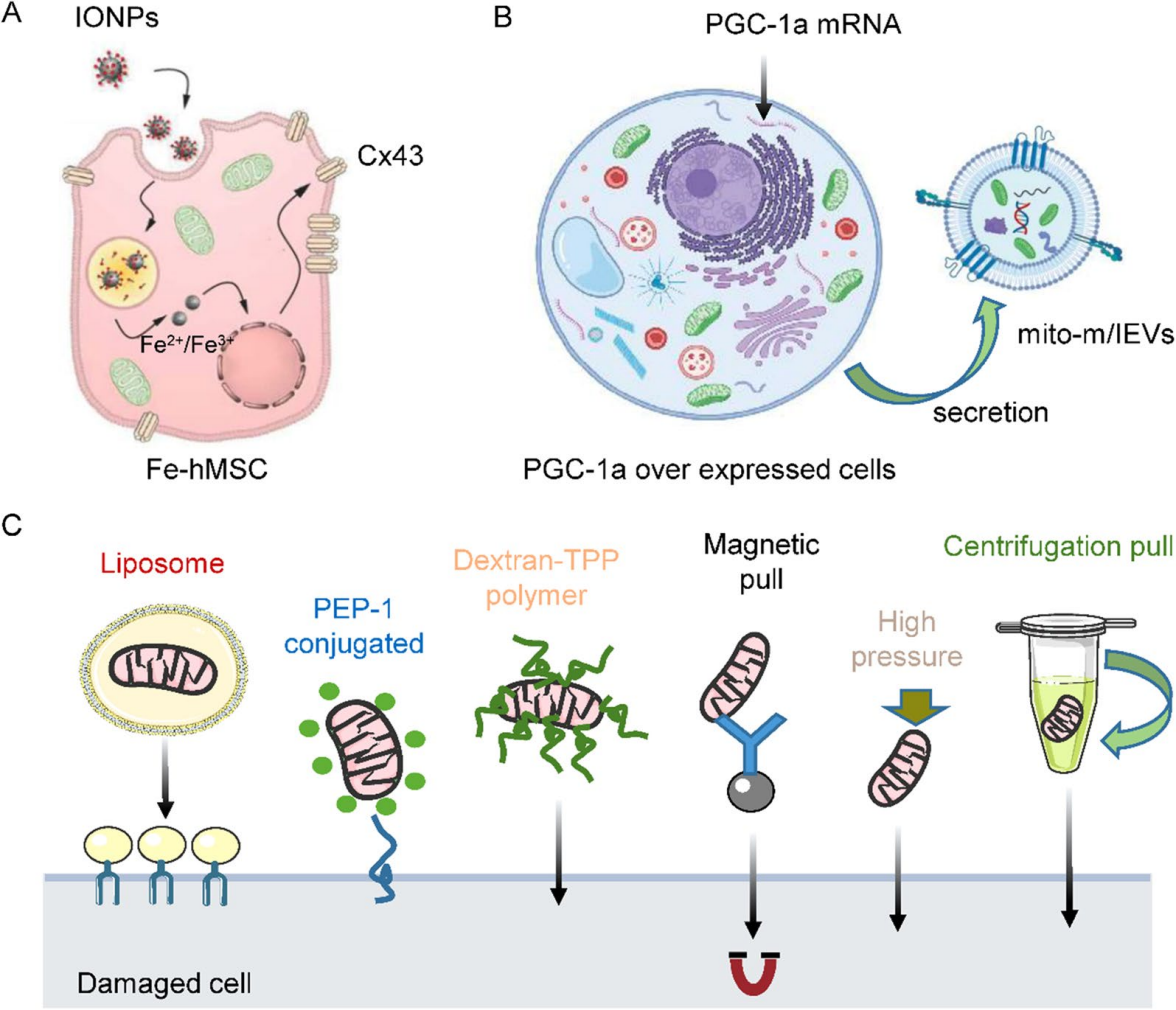
Representation of mitochondrial-internalization mechanisms used in mesotherapy. **A** Schematic illustration showing the potential strategy of using IONPs to augment intercellular mitochondrial transfer from hMSCs. **B** Increased mitochondrial biogenesis in PGC-1a–activated human brain ECs might permit the engineering of EVs with a higher mitochondrial load-m/IEVs (mitochondrial/IONPs-EVs). **C** Techniques developed to improve exogenous-mitochondrion internalization

MRT has proven to be a potential therapy for improving mitochondrion-related diseases. To effectively treat diseases in humans using MRT, various strategies such as peptide-mediated mitochondrial delivery (PMD)_,_ magnetic nanoparticles (NPs)_,_ centrifugation-based methods, iron oxide NP (IONP)–engineered human MSCs (hMSCs), mediated mitochondrial transfer, and medium-to-large EVs (m/LEVs) have been developed and applied (Fig. 4).

Isolated functional mitochondria can selectively replace damaged mitochondria and have been used to treat mitochondrial diseases. In 2017, Boston Children’s Hospital (Boston, MA, USA) was the first institution to use autologous mitochondria transfer in the clinical treatment of myocardial I/R injury in children [9]. Mitochondria isolated from the patient’s non-ischemic skeletal muscle were injected directly into the damaged myocardium; improvements in ventricular function were observed without adverse complications such as arrhythmia, intramyocardial hematoma, or scarring [9]. In the second clinical trial, initiated at Sun Yat-sen University (Guangzhou, China) in 2018, mitochondria were autologously microinjected from BM-MSCs into human sex cells (oocytes and sperm; ClinicalTrials.gov No. NCT03639506) to improve oocyte quality. Nevertheless, the internalization of isolated mitochondria was decreased in target cells due to the negative surface charge. In response, PMD [152–154]_,_ magnetic NPs [155], and centrifugation-based methods [156] were applied to improve the efficiency of naked-mitochondrial transplantation. Recently, biocompatible polymers have been proposed as a more efficient strategy for the delivery of isolated mitochondria to enhance target cell internalization [157].

MSC transplantation is considered an efficient means of mitochondrial delivery. Currently, trials for clinical cell therapies are ongoing for mitochondrial-related diseases that include Pearson syndrome (No. NCT03384420), ophthalmic pathology (including age-related macular degeneration and glaucoma; No. NCT03011541), and inherited metabolic disorders (including mitochondrial neurogastrointestinal encephalopathy; No. NCT02171104). However, the clinical application of MSCs is limited by oncogenicity, abnormal differentiation, and vascular occlusion [158]. A recent study reports using IONPs to efficiently and safely transfer mitochondria from hMSCs to damaged cells, which could restore mitochondrial bioenergetics of damaged cells. Ionized IONPs promote the formation of C×43-containing GJCs (C×43-GJCs), which selectively promote the transfer of hMSC mitochondria to damaged cells. In a mouse model of pulmonary fibrosis, IONP-engineered hMSCs promoted intercellular mitochondrial transfer and significantly alleviated fibrosis progression without any safety concerns [159].

EVs are natural cell-derived drug carriers that can carry a variety of bioactive components of lipids, proteins, and nucleic acids for intercellular communication. EVs of particle size < 200 nm are called small EVs (SEVs), and those of particle size > 200 nm are called m/LEVs; the latter can naturally transport mitochondria during biogenesis. A few reports suggest that m/LEVs enhance the survival of damaged recipient tissues by participating in the transfer of healthy mitochondria, resulting in a functional increase in cellular/tissue ATP levels and cellular bioenergetics [17, 24, 27]. The maintenance of naked mitochondrial structure in serum is difficult, but m/LEVs can effectively protect the integrity and functional activities of mitochondria and prolong their lifespan in blood, making them a promising carrier for MRT. A recent study reports that m/LEV can be engineered with an abundant mitochondrial load by activating PGC-1α [160], but such trials are often very expensive and time-consuming. Research on expanding EV production capacity and m/LEV collection rate with rich mitochondrial load could help broaden clinical applications of mitochondrial therapy.

MRT strategies for systemic administration of isolated mitochondria, stem cells, or EVs have poor specificity, which affects blood cells and vessel-rich organs such as the lungs and liver. Therefore, the efficacy of MRT benefits from the improvement of the targeted delivery and internalization efficiency of mitochondria to specific tissues or organs. We believe that the main focus of future research should be to develop carriers for specific cell delivery or to overcome the challenges of mitochondrial-internalization efficiency.

## Conclusions

This review provides a comprehensive description of the current progress of research in mitochondrial replacement transplantation for metabolic diseases and discusses the ethical, immunogenic, storage, and targeted-delivery issues that challenge MRT. To develop better mitochondrial-transplantation products for the clinical treatment of metabolic diseases, we must establish an ideal MRT system.

Because mitochondria are easily obtained from cultured cells and the technology of mitochondrial isolation and preservation is becoming more mature, large-scale mitochondrial-donation centers are expected to be established in the future; thus, when autologous transplantation cannot be performed, a suitable donor can be found in time. However, whether MRT is feasible in specific indications remains unclear. Therefore, a skin allograft rejection and tolerance test should be performed to ensure the safety of MRT, and the subsequent dosing regimen and route of administration should be based on the combined onset characteristics of the disease. In acute situations such as infarction, rapid administration of successfully matched healthy mitochondria can ameliorate mitochondrial dysfunction and subsequent cell death, while other chronic mitochondrial dysfunction diseases might require continuous intermittent injections of mitochondria. Nevertheless, doses of mitochondrial and ectopic implantation in healthy tissue could lead to side effects. Real-time monitoring of the therapeutic process is equally important. The addition of imaging tools such as biodegradable fluorescent probes or quantum dots in mitochondrial-delivery vehicles might be beneficial. The development of MRT technologies could offer new therapeutic options for mitochondrial transplantation. In conclusion, although MRT faces many problems and challenges, it still has great development prospects and a good clinical market.

## Data Availability

Not applicable.

## Abbreviations

NAFLD: Nonalcoholic fatty liver disease
IR: Insulin resistance
TNTs: Actin-based tunneling nanotubes
EVs: Extracellular vesicles
T2D: Diabetes mellitus
mROS: Mitochondrial oxidase-derived ROS
NASH: Nonalcoholic steatohepatitis
ETC: Electron transport chain
MSCs: Mesenchymal stem cells
STZ: Streptozotocin
Mt: Mitochondria
PGC-1α: Receptor-activated receptor gamma-coactivator-1α
Mito-Plt: Platelet-derived mitochondria
MRTs: Mitochondria Transfer
DAMPs: Damage-associated molecular patterns
Cyt C: Cytochrome C
IONPs: Iron oxide nanoparticles
